# High-Throughput Sequencing of Environmental DNA as a Tool for Monitoring Eukaryotic Communities and Potential Pathogens in a Coastal Upwelling Ecosystem

**DOI:** 10.3389/fvets.2021.765606

**Published:** 2021-11-03

**Authors:** Raquel Ríos-Castro, Alejandro Romero, Raquel Aranguren, Alberto Pallavicini, Elisa Banchi, Beatriz Novoa, Antonio Figueras

**Affiliations:** ^1^Inmunology and Genomics, Marine Research Institute (IIM-CSIC), Vigo, Spain; ^2^Department of Life Sciences, University of Trieste, Trieste, Italy; ^3^Division of Oceanography, National Institute of Oceanography and Applied Geophysics, Trieste, Italy

**Keywords:** metabarcoding, eukaryote, high-throughput sequencing, eDNA, environment, pathogens

## Abstract

The marine environment includes diverse microeukaryotic organisms that play important functional roles in the ecosystem. With molecular approaches, eukaryotic taxonomy has been improved, complementing classical analysis. In this study, DNA metabarcoding was performed to describe putative pathogenic eukaryotic microorganisms in sediment and marine water fractions collected in Galicia (NW Spain) from 2016 to 2018. The composition of eukaryotic communities was distinct between sediment and water fractions. Protists were the most diverse group, with the clade TSAR (Stramenopiles, Alveolata, Rhizaria, and Telonemida) as the primary representative organisms in the environment. Harmful algae and invasive species were frequently detected. Potential pathogens, invasive pathogenic organisms as well as the causative agents of harmful phytoplanktonic blooms were identified in this marine ecosystem. Most of the identified pathogens have a crucial impact on the aquacultural sector or affect to relevant species in the marine ecosystem, such as diatoms. Moreover, pathogens with medical and veterinary importance worldwide were also found, as well as pathogens that affect diatoms. The evaluation of the health of a marine ecosystem that directly affects the aquacultural sector with a zoonotic concern was performed with the metabarcoding assay.

## Introduction

Marine ecosystems harbor highly diverse eukaryotic microorganisms, many of which are unknown or ignored due to difficulties in culturing and identifying them. Biodiversity in coastal marine environments is endangered due to climate change and human activity, and many species could disappear without being identified (1). Many marine eukaryotic species remain to be described, with the proportion of unknown diversity ranging from 24 to 98% depending on the taxonomic group (2). For example, the small eukaryotes (<1 mm) that play important ecological roles in aquatic ecosystems remain poorly described (3). In this context, the study of the taxonomic and functional diversity of small eukaryotic organisms in marine ecosystems is being enriched by incorporating novel molecular tools (2, 4) that complement conventional methods based on the morphological and nutritional characterization of specimens *in vivo*, light and fluorescence microscopy, electron microscopy, or liquid chromatography (HPLC) (5, 6), improving our knowledge on their ecological role in ecosystem functioning (7).

DNA metabarcoding technology allows the identification of thousands of species at the same time by sequencing the DNA extracted from organisms or environmental DNA (eDNA) in water or sediments on a high-throughput sequencing (HTS) platform (8, 9). Initially, this technology was used for the characterization of marine prokaryotes (10, 11). Subsequently, studies were also focused on eukaryotic diversity characterization, including metazoans, plants, fungi, and protists. In the marine environment, benthic and pelagic organisms have been described from the sediment (12–14) and water column (14–17), permitting the detection of new or rare taxa in the ecosystem (3, 18). DNA metabarcoding has also been used to detect exotic marine species (16), helping to establish early biosecurity alerts (19).

Protists constitute the bulk of eukaryotic diversity in marine communities (20) and play a variety of crucial roles in aquatic ecosystems, acting as photosynthesizers (primary producers), heterotrophs (predators and parasites) and mixotrophs (3, 6). Moreover, many protists, together with fungi or even metazoans, can act as potential pathogens affecting organisms and ecosystems and have been implicated in global-scale declines in a wide range of marine and terrestrial species (21–25).

Total protist diversity is usually analyzed by using the V9 and V4 regions of the 18S rRNA gene (26, 27). The V9 region is widely used in HTS platforms due to its short length (180 bp), although it provides lower phylogenetic information than the V4 region (400–500 bp length). In contrast, V4 is not adequate for Illumina platforms, and amplicon length variability could produce PCR or sequencing biases. Organisms belonging to the Alveolata group have usually remained unexplored using V4 but have been detected using V9. Thus, it seems that V9 reveals much better resolution in most protistan supergroups than V4 (28).

Abiotic and biotic factors (solar irradiation, nutrients, temperature, or predation) influence the dynamics of the eukaryote community (29). Moreover, wind-driven upwelling and downwelling events that cause alternation between water column stratification and mixing (30) induce seasonal succession of small planktonic eukaryotes in surface waters (31). In particular, the Ría de Vigo (NW, Spain) is highly influenced by the upwelling dynamics which occurs mainly in summer periods, when oceanic currents from the depths enter in zone (32). However, high-throughput sequencing technology only has been used to study seasonal succession of small-eukaryote community in surface waters (31) and co-ocurrence networks based on eukaryotic and prokaryotic associations were performed (33).

The area of study has been the scenario of the detection of Toxic Harmful Algae Blooms (THAB), invasive species and pathogens that affects the aquaculture sector. Toxic algae generate toxins that causes severe impact in the aquaculture sector and human heath by the consumption of contaminated bivalves. The importance of fish and shellfish aquaculture in the area makes that the control of pathogens is a priority. Several parasites associated with bivalve mortalities such as *Perkinsus olseni* (34) or different species of *Marteilia* (35, 36) have been detected near of Ría de Vigo (37, 38). Mortalities of turbot *Scophthalmus maximus*, one of the most fish farmed species have been registered due to the parasite *Philasterides dicentrarchi* in farms of Galicia (39, 40). These studies on the detection of pathogens have been made by histopathology, morphological studies and real-time PCR assay and, until now, metabarcoding and high-throughput sequencing have not been used before in Ría de Vigo.

Human sewage and run-off from farms are released through sewage disposal systems, reaching the marine environment (41). In this context, DNA metabarcoding technology can also detect the presence of these pathogens and could be used to predict the ecological conditions of the marine ecosystem (42). However, this technology requires the construction of a robust reference database and the use of highly efficient primers for the correct amplification of the target gene without missing any taxon information (4, 8).

In this study, we evaluated the use of DNA metabarcoding technology to describe eukaryote biodiversity in a highly productive coastal marine ecosystem. In addition to identifying non-indigenous species and harmful algae, our objective was to detect potential pathogens for both marine cultured species and for relevant species in the ecosystem.

## Materials and Methods

### Sampling

**Figure 1:**
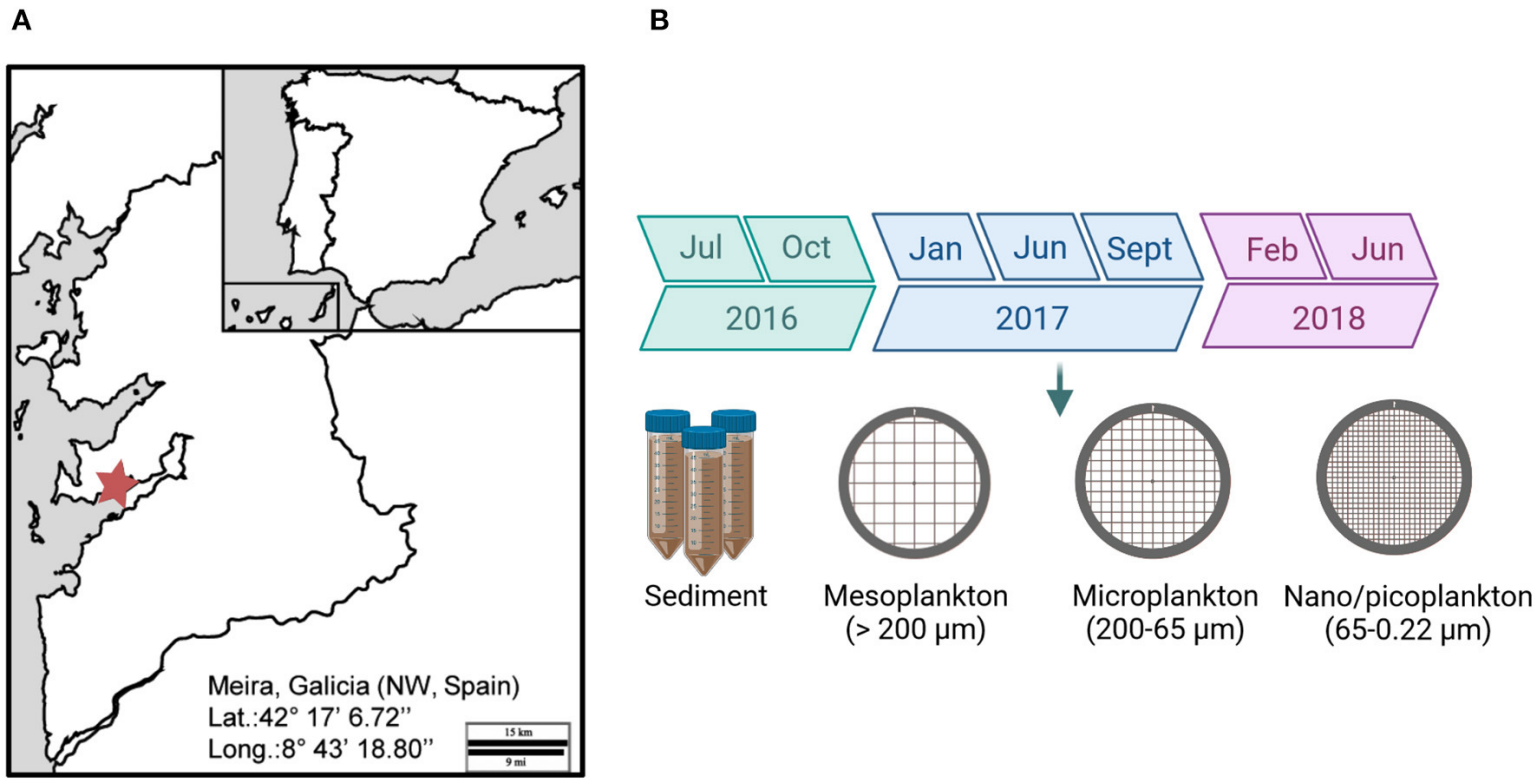
**(A)** Location of the bivalve production area in the Ría of Vigo (Meira, NW, Spain). **(B)** The sampling period extended from 2016 to 2018. Environmental samples were collected for analysis.

Eukaryotic diversity was evaluated in water and sediment from a bivalve production area located in the Ría de Vigo, NW Spain (Meira: 42°17′6.72″ N/8°43′18.80″ W) (Figure 1A). The sampling period was from summer 2016 to summer 2018. Samples were collected every 3 months (Figure 1B). Superficial sediment was collected and kept at −20°C. Seawater was successively filtered by different pore sizes. A volume of 75 m^3^ of seawater was filtered with a 200 μm plankton net (mesoplankton). A volume of 40 L was filtered again through a 65 μm pore size net (microplankton), and finally, 2 L of seawater was filtered by using a 0.22 μm pore size (nanoplankton-picoplankton). The nomenclature of the water fractions was adapted from Sieburth et al. (43). Filters were also kept at −20°C until use.

### DNA Extraction, Amplification, and Sequencing

A total of 24 samples of sediment and water fractions were processed. DNA from the sediment and water was isolated using the PowerSoil DNA isolation Kit (Qiagen, Düsseldorf, Germany) following the manufacturer's protocol.

The V9 region of the 18S rDNA (180 bp long) was amplified using the universal eukaryotic-specific primers 1380F/1510R described by Amaral-Zettler et al. (26). Amplicons were purified using the Qiaquick PCR purification kit (Qiagen) and quantified. A library for 18S metagenomic sequencing was prepared using the Herculase II Fusion DNA Polymerase Nextera XT Index Kit V2 (Illumina), and paired-end sequencing (2 x 300) was performed on an Illumina MiSeq platform (Macrogen, Korea). The raw read sequences obtained were deposited in the Sequence Read Archive (SRA) (http://www.ncbi.nlm.nih.gov/sra) under the BioProject accession number PRJNA761019.

### Bioinformatic Analysis

A custom 18S eukaryotic reference database was previously constructed to classify metagenomic reads assigning an operational taxonomic unit (OTU) to the sequences obtained after the Illumina assay. Briefly, a total of 726,478 sequences ranging from 100 to 10,000 bp in length were downloaded from the NCBI database. Sequences from bacteria, unverified organisms, environmental samples, and predicted sequences were excluded. Their taxonomic information was assigned using the Python script “entrez_qiime.py” (44), and the non-annotated sequences showing 99% coverage and 100% similarity were subjected to additional BLAST analysis against the nucleotide collection nr/rt to complete the taxonomy information. A total of 723,146 sequences (99.54%) were taxonomically classified.

The CD-HIT_Est tool from the CD-hit package 4.6.8 (45) was used to cluster the sequences with 99% coverage and 100% similarity. Briefly, sequences were first sorted in order of decreasing length. The longest sequence became the representative sequence of the first cluster, which included all sequences with 99% similarity and 90% coverage. When the similarity of the sequence with the representative sequence was below 99%, a new cluster was defined with that sequence as the representative. CLC workbench 12 was used to merge the taxonomy of the database with the representative sequence of each cluster and their accession number.

#### Database Annotation, Trimming, and Clustering

The Microbial Genomics Module of Qiagen CLC Workbench 12 was used for data analysis. Paired-end reads were trimmed using the sequences of the primers (1380F and 1510R). Low-quality reads were also trimmed by quality scores (limit 0.05 = minimum average quality score 20), by the number of ambiguous nucleotides (maximal 2 ambiguous nucleotides), and by the length of the primers (below 100 and above 200 bp).

Paired reads were then merged and grouped using the OTU-clustering tool. The similarity threshold was set at 94% for 18S reference-based OTU clustering and 80% for *de novo* OTU clustering. Finally, one representative sequence of each OTU was assigned to the best match in the reference database. Singletons and chimeras were excluded from the analysis. Pathogens sequences were further verified by a Blast confirming their identity with a 100% of similarity in the NCBI database.

#### Alpha and Beta Diversity

Alpha diversity evaluates how many different species can be detected in a microbial ecosystem by the number of different OTUs and how they are distributed within a community (46). Alpha diversity was estimated by constructing rarefaction curves calculated by subsampling OTU abundances in the different samples at different depths. Samples were rarefied at the minimum sample read depth for each amplicon. The alpha diversity was also evaluated by measuring the relative abundance (percentage of reads associated with each OTU or taxon) and richness (number of OTUs or taxa included in each sample) in all samples.

Beta diversity shows the differences between microbial communities from different environmental samples, focusing on the difference in their taxonomic abundance profiles (47). Beta diversity analysis was performed by calculating Bray–Curtis distances between each pair of samples and applying principal coordinate analysis (PCoA) on the distance matrices. Permutational multivariate analysis of variance (PERMANOVA) based on the Bray-Curtis dissimilarity index was also estimated.

## Results

### Sequencing Data

After sequencing and trimming, a total of 4,034,813 reads were obtained from environmental samples (sediment and water) (Table 1) with an average length of 146 nucleotides. A detailed description of the number of sequences obtained in each step of the analysis is specified in Table 1 and Supplementary Tables 1–3. The OTU-clustering process generated 7,241 OTUs using a total of 1,729,005 filtered reads. To decrease the number of non-representative taxa, OTUs with a combined abundance in all samples ≤ 5 were excluded from the analysis. A total of 4,069 OTUs were finally selected and used for the following analyses.

**Table 1:** Number of reads and OTUs obtained through the analysis of data obtained using Illumina.

**Sequence description**	**Environmental samples (*N* = 24)**
Raw reads	4,683,658
Reads after trimming	4,034,813
Number of merged reads	1,979,038
Filtered or chimaeric reads	250,033
Reads in OTUs	1,729,005
Total predicted OTUs	7,241
OTUs based on database	2,696
*De novo* OTUs	4,545
OTUs combined abundance > 5	4,069
Reads in OTUs >5 combined abundance	1,720,753

*The number of reads before and after the trimming and clustering procedure is specified. Number of OTUs based on the database and de novo OTUs obtained after the clustering procedure*.

### Overview of Eukaryotic Diversity

**Figure 2:**
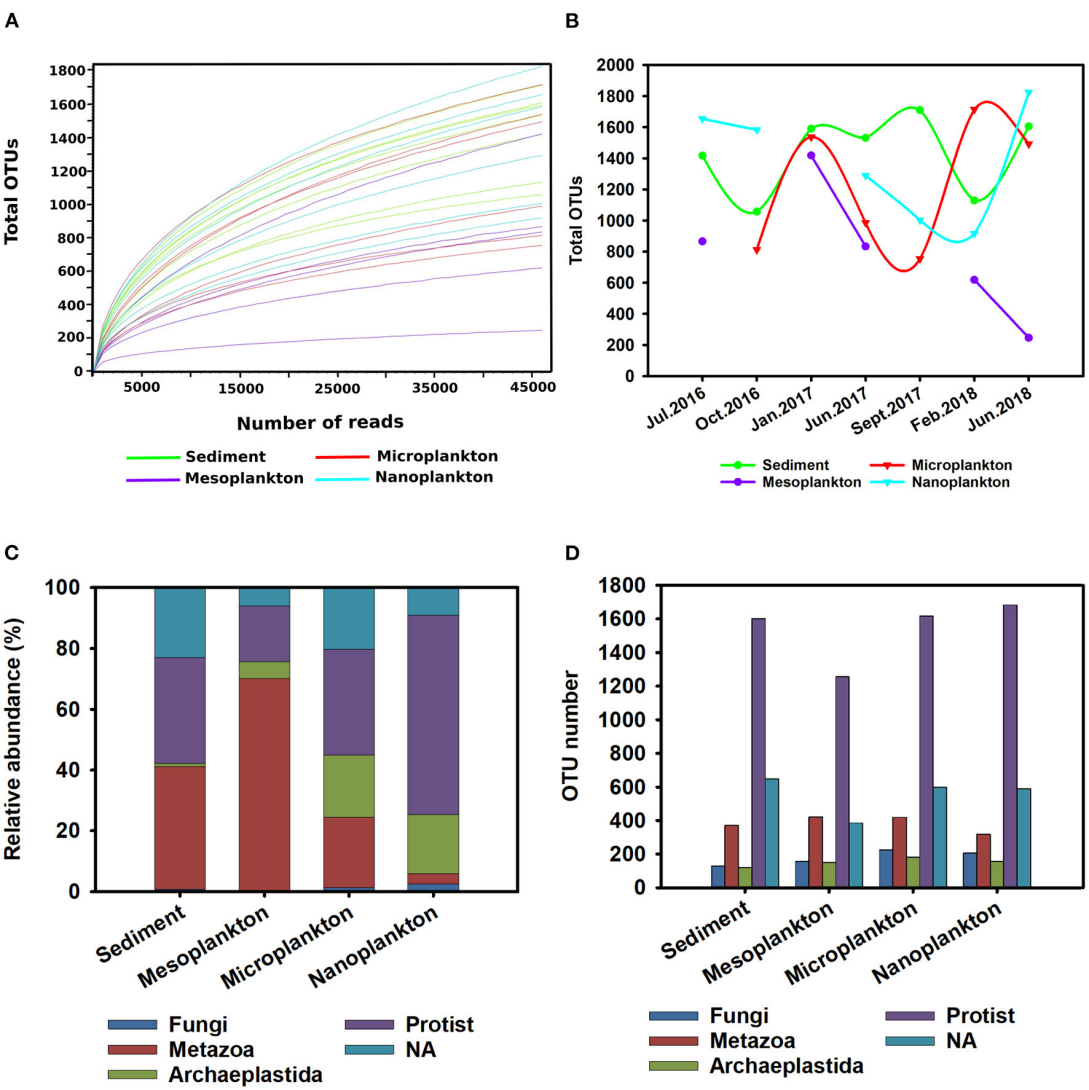
Overview of eukaryotic diversity. **(A)** Rarefaction curves constructed to analyse the alpha diversity in the environmental samples. A subsampling depth of 46,108 reads was used. **(B)** Seasonal variations in the alpha diversity registered during the sampling period. **(C,D)** General description of eukaryotic diversity [protist, metazoan, Archaeplastida, Fungi, and non-classified eukaryotes (NA group)] presented in the environment. The relative abundance (percentage) of the different taxonomic groups in environmental samples **(C)** is shown. The richness of the different taxonomic groups based on the number of OTUs included in each group was analyzed **(D)**.

The number of different taxonomic groups and their distribution within the ecosystem were evaluated by alpha diversity. Rarefaction curves were constructed with a subsampling depth of 46,108 reads, corresponding to the sample with the lowest number of reads. The curves show that the subsampling sequencing effort successfully represented a correct total diversity in the environmental samples (Figure 2A). Seasonal variations in alpha diversity were observed. In addition, the diversity of eukaryotes in sediment was almost constant during the sampling period, and significant seasonal variations were observed in the water. In the meso- and microplankton, the number of OTUs was low during summer and increased in winter. Opposite kinetics were observed in the nanoplankton, where the highest diversity was detected during summer and decreased in winter (Figure 2B).

The most abundant taxonomic groups were metazoans and protists. The metazoan group was the largest (34%) composing 40 and 70% of the total abundance of sediment and mesoplankton, respectively. In contrast, the protist group was predominant in the nanoplankton (65%) (Figure 2C). The protists represented 38.56% abundance in the environment and 52% of environmental richness with the highest number of OTUs, followed by the unclassified eukaryotes (NA group) (20.54% of total OTUs). Thus, the high number of unknown eukaryotes present in the marine environment was confirmed. The metazoan group included 300–400 OTUs, representing 15.61% of the total OTUs, while Archaeplastida and Fungi included the lowest number of OTUs (264 and 206, respectively), which barely represented 5% of the total OTUs (Figure 2D).

**Figure 3:**
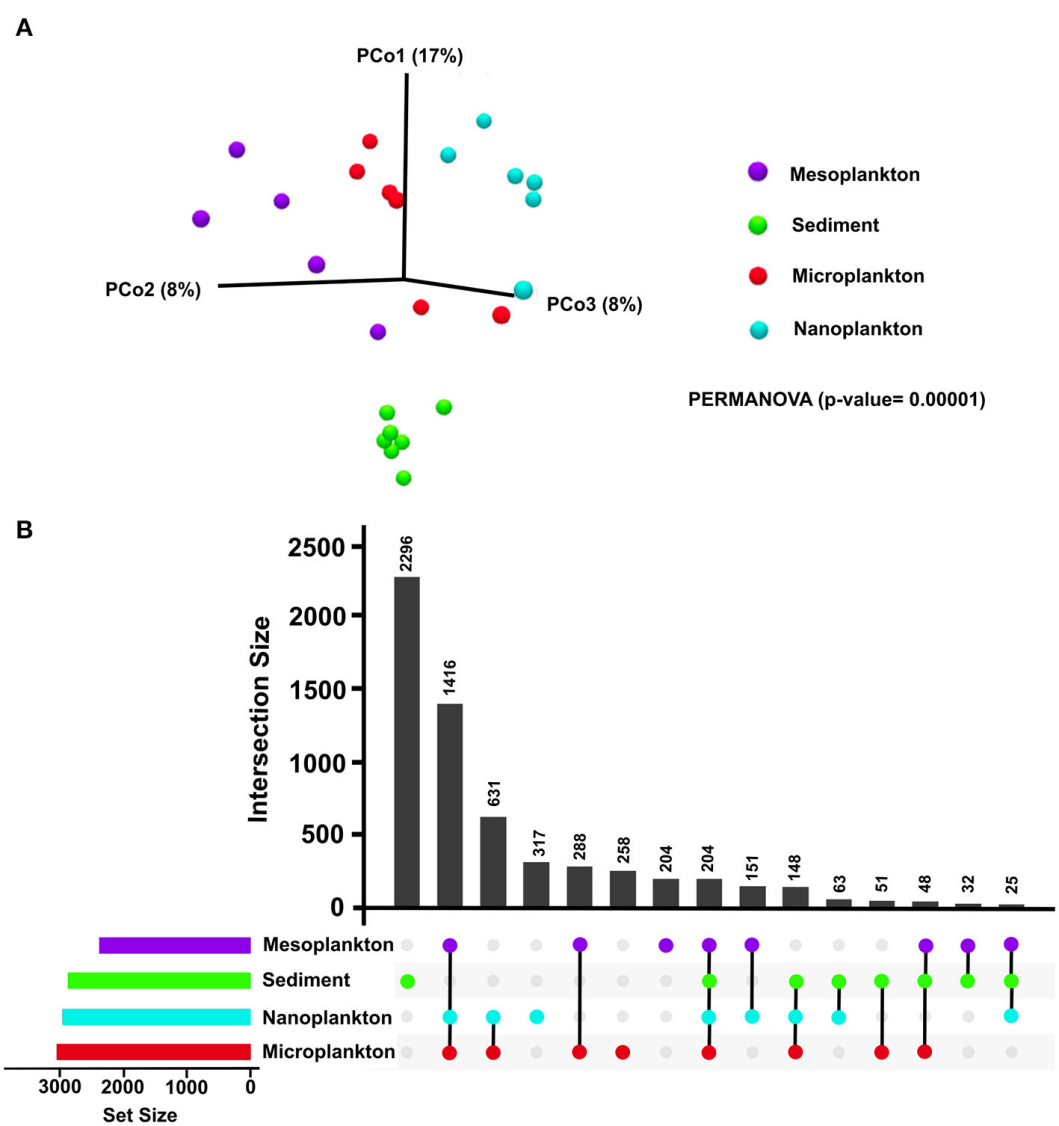
**(A)** Beta diversity analysis of each environmental compartment (sediment, mesoplankton, microplankton, and nanoplankton). Statistical differences were calculated using the pseudo f statistic (2.51) and a *p* = 0.00001. **(B)** Representation of the number of common and exclusive OTUs detected in sediment and water. Horizontal bars represent the total number of input OTUs in each environmental compartment.

Significant differences in the composition of eukaryotic communities among environmental compartments were clearly observed by beta diversity analysis. Although no significant differences were observed between the water fractions in pairwise comparisons, the sediment was clearly clustered and separated from the meso-, micro-, and nano-plankton fractions (Figure 3A). Exclusive OTUs were obtained from each environmental compartment. Sediment showed 2,296 exclusive OTUs (5,644% total OTUs) but also in nanoplankton (317), microplankton (258) and mesoplankton (204). Only 204 OTUs where shared among all environmental compartments (5% total OTUs) (Figure 3B).

### Diversity of Eukaryotes in the Marine Ecosystem

A detailed description of the different eukaryotic groups present in water and sediment is described below. Special attention was given to the protist group since it is the most diverse group in the marine environment.

#### Archaeplastida

The Archeplastida group comprises red and green macroalgae, land plants, and some groups of small unicellular algae. This kingdom represented 20% of the total abundance in micro- and nanoplankton. At the phylum level, Rhodophyta and Chlorophyta were the dominant groups among the micro- and nanoplankton. The 15 most abundant genera are represented in Table 2.

**Table 2:** Top 15 most abundant Archaeplastida genera detected in sediment and water fractions.

**Phylum**	**Genus**	**Sediment**	**Water**	**Meso-**	**Micro-**	**Nano-**
Rhodophyta	*Melanothamnus*	2,785	97,139	8,686	56,056	32,397
	*Polysiphonia*	1,470	39,191	4,261	17,047	17,883
	*Ceramium*	558	5,665	1,773	1,820	2,072
	*Pyropia*	76	2,705	396	594	1,715
	*Callithamnion*	20	1,861	331	1,382	148
	*Acrosymphytales* sp.	6	1,182	1,021	159	2
Chlorophyta	*Tetraselmis*	1,104	18,611	77	1,376	17,158
	*Micromonas*	0	4,522	12	11	4,499
	*Ostreococcus*	8	3,319	40	23	3,256
	NA Chlorophyta	268	1,026	8	365	653
	*Ulva*	74	646	422	202	22
	*Mantoniella*	10	592	2	31	559
Streptophyta	*Phyllospadix*	58	7,162	3,376	3,643	143
	*Salix*	0	1,687	222	1,452	13
	*Alnus*	6	808	113	689	6

*Absolute abundance in each environmental compartment is presented. Meso, micro, and nano represent mesoplankton, micropankton, and nanoplankton, respectively*.

#### Fungi

**Figure 4:**
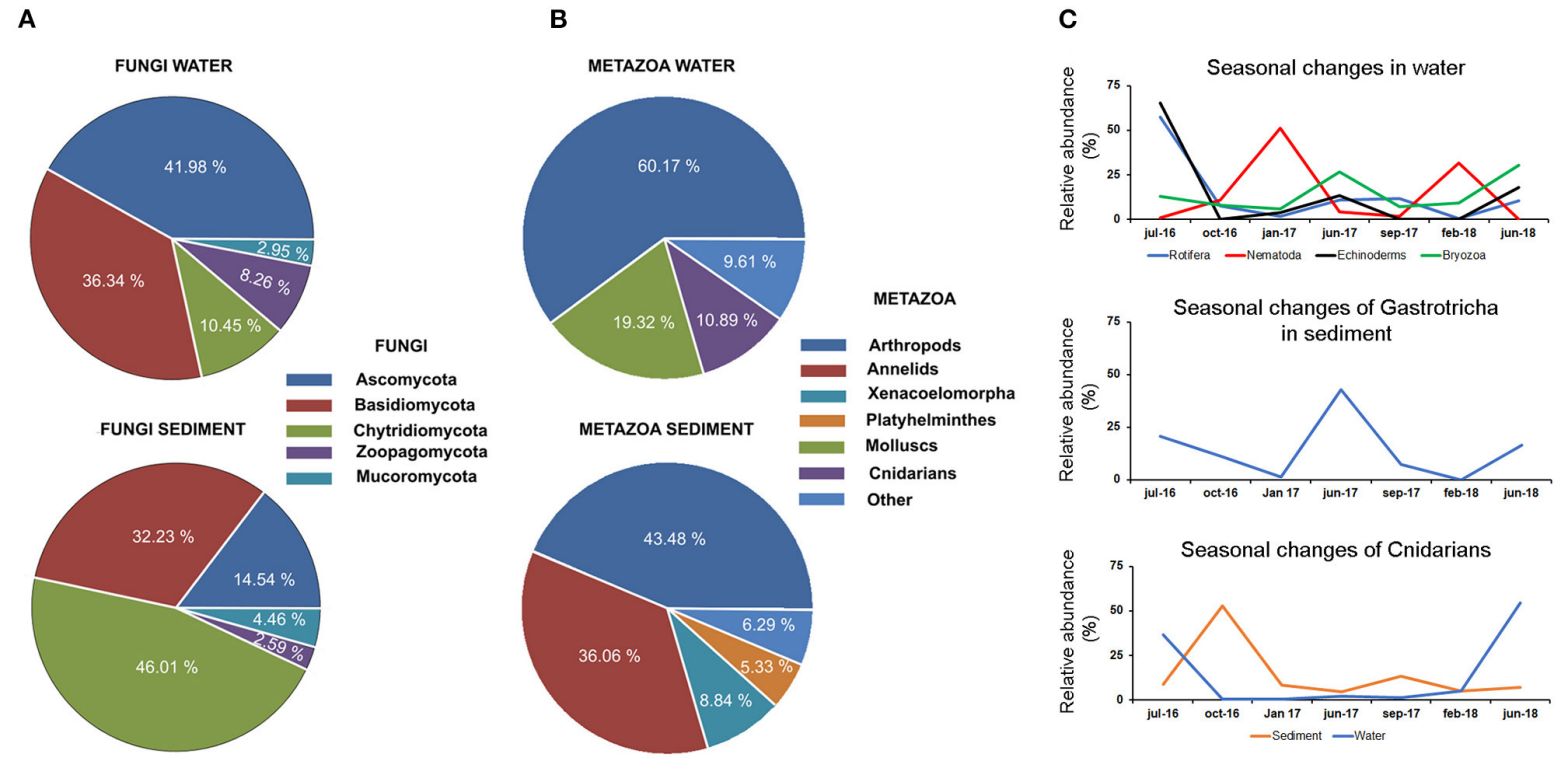
Distribution of fungi **(A)** and metazoan **(B)** in the water and sediment. **(C)** Temporal data allowed us to describe seasonal changes in the abundance of several taxonomic groups in water and sediment. Seasonal changes were also described for cnidarians in water and sediment.

Fungi was the least abundant group (<2% of the total abundance in the environment). The phylum Ascomycota (42%) and the phylum Chrytridiomycota (46%) were the most abundant phyla in the water and sediment, respectively. The phylum Basidiomycota was present in both water and sediment at high abundance (32%) (Figure 4A).

#### Metazoans

The abundance of metazoans in the water was ~60% higher than that observed in the sediment (Figure 4B). The eukaryotic communities found in the water were dominated by arthropods and molluscs, which represented 60 and 20%, respectively, of total metazoans. Planktonic forms of cnidarians constituted 10% of the total abundance. Chordates were mainly represented by fish species (clupeids), which were only present in the water fraction (2.5% of total abundance) (Figure 4B). The distribution of metazoans in the sediment was completely different, being mainly composed of arthropods (43%) and other kinds of worms, such as annelids and platyhelminths (41%) (Figure 4B). Temporal data allowed the description of seasonal changes in the abundance of several metazoan groups (Figure 4C). A high abundance of several taxonomic groups was observed during the summer period, followed by a decrease in autumn and winter. This pattern was observed in cnidarians, rotifers, echinoderms, and bryozoans detected in the water and in the Gastrotricha group in the sediment. In contrast, the nematodes increased in the sediment during winter. In the cnidarian group, the decrease in swimming forms after summer was followed by an increase in benthonic stages in the sediment (Figure 4C).

The analysis of the metazoan group also revealed the presence of four alien or invasive species in the environment (Table 3). Their potential invasiveness was scored following Tsiamis et al. (48) based on the likelihood of arrival, establishment, spread, and potential impact with a maximum value of 48. The bivalve mollusc *Xenostrobus securis*, which had the highest score (48), was detected in all environmental compartments at high abundance. The invasive arthropod *Pseudodiaptomus marinus* was only present in water fractions. The ascidian *Microcosmus squamiger* was also detected in all environmental samples (Table 3).

**Table 3:** List of invasive species detected in environmental samples.

**Invasive specie**	**Taxonomy**	**Sediment**	**Water**	**Score**
*Xenostrobus securis*	Mollusca, Bivalvia	29	1,763	48
*Pseudodiaptomus marinus*	Arthropoda, Hexanauplia	0	2,501	45
*Microcosmus squamiger*	Chordata, Ascidiacea	2	20	42

*The total abundance in sediment and water is presented. Their potential invasiveness score, according to Tsiamis et al. (48), is also presented. Organims were confirmed with a 100% similarity with the NCBI database*.

#### Protists

**Figure 5:**
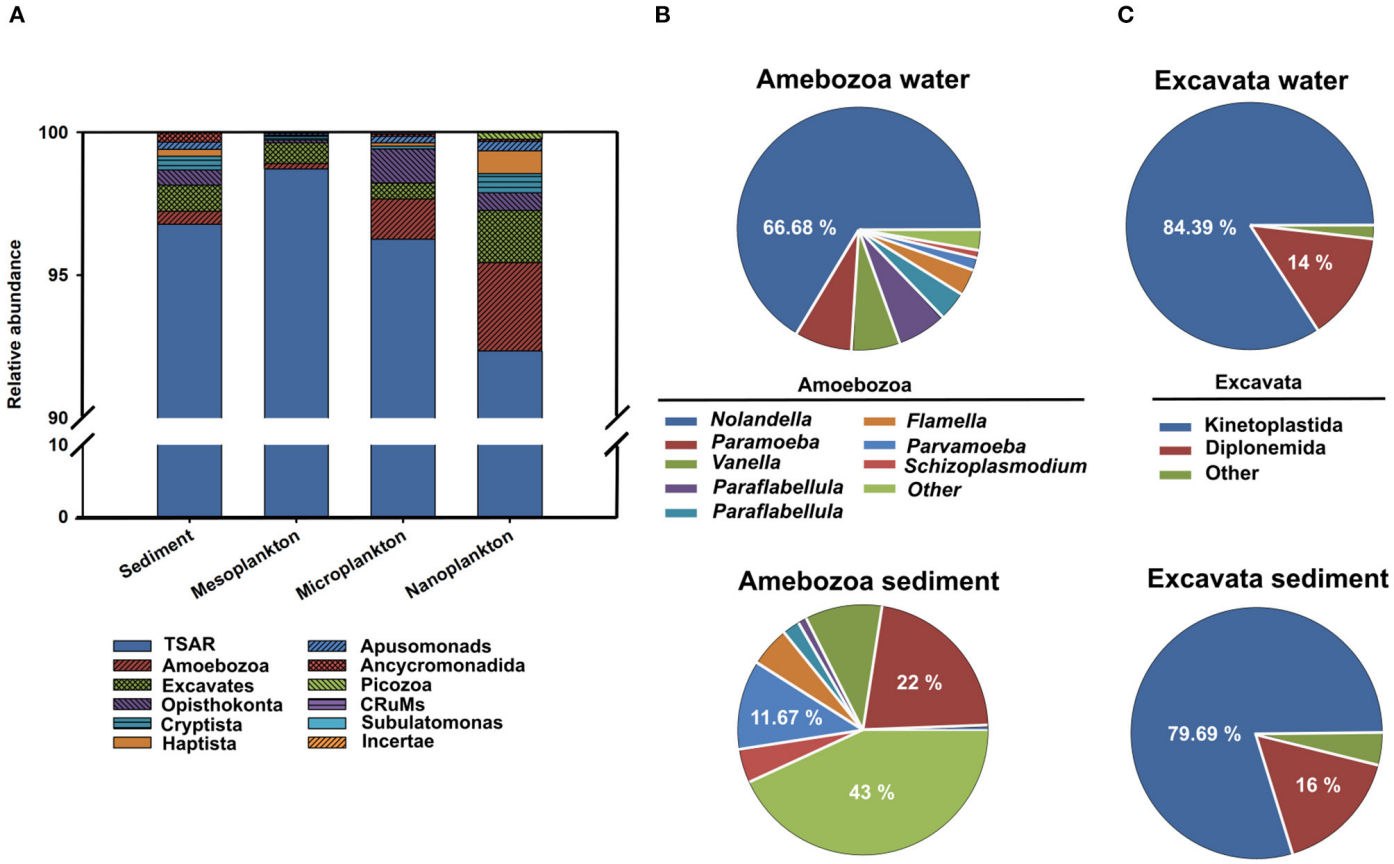
The presence of protists classified according to Burki et al. (49) into four supergroups (Amebozoa, Excavata, TSAR, and a fourth group including other minor organisms) is represented. **(A)** Relative abundance of the different supergroups in sediment, meso-, micro-, and nanoplankton. Relative abundances of Amebozoa **(B)** and Excavata **(C)** in sediment and water.

The majority of eukaryotes found in the environment belonged to the protist group. A total of 663,556 reads were clustered in 2,127 OTUs. Protists were classified according to Burki et al. (49) into different supergroups, including Amebozoa, Excavata, TSAR (Telonemia, Rhizaria, Alveolata, and Stramenopila) and a fourth group including other minor organisms. In general, the supergroup TSAR was dominant in the environment, representing more than 90% of all protists. Amoebozoa and Excavata constituted <5% of the protist abundance (Figures 5A–C).

**Figure 6:**
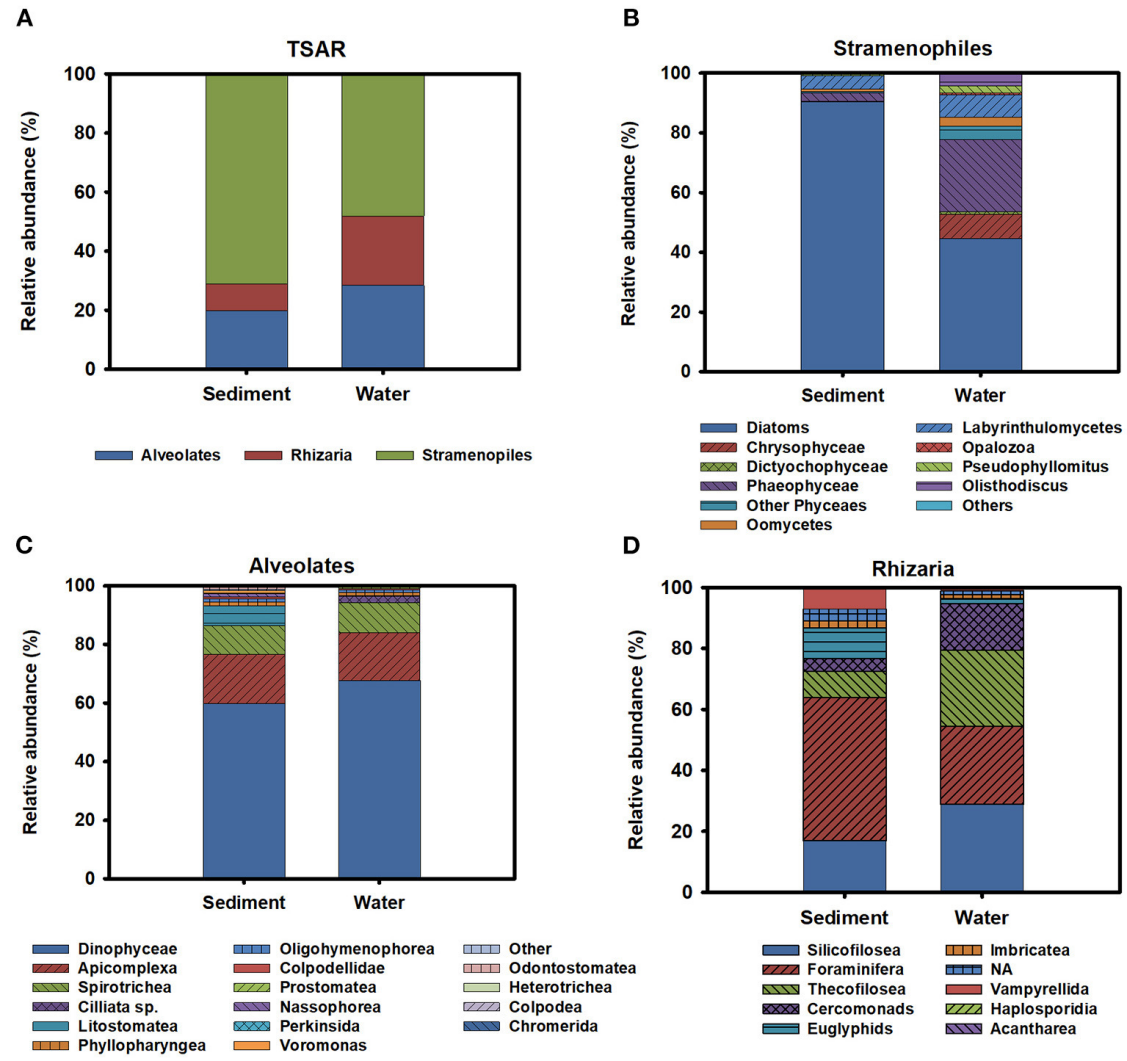
**(A)** General description of the diversity observed inside the TSAR supergroup. Relative abundance of the different members of Stramenopiles **(B)**, Alveolates **(C)**, and rhizarians **(D)** in sediment and water.

**Figure 7:**
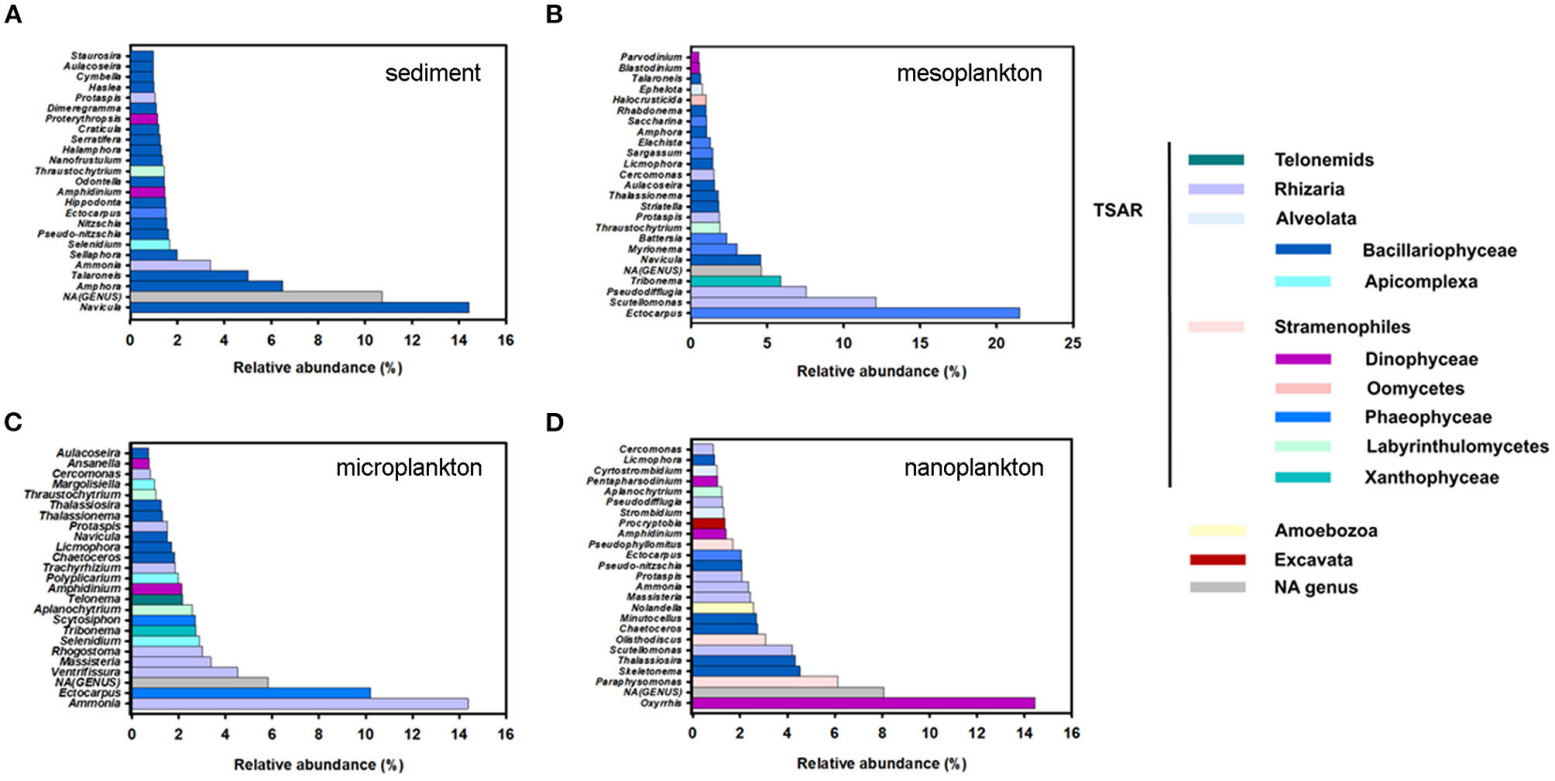
Top 25 most abundant genera of protists in sediment **(A)**, mesoplankton **(B)**, microplankton **(C)**, and nanoplankton **(D)**. Genera were classified in the legend according to their pertinence of higher taxonomic groups, mainly TSAR, Amoebozoa, and Excavata.

TSAR supergroup was the most abundant group of protists in all environmental compartments. Stramenopiles were more abundant in the sediment and represented 70% of the TSAR abundance. Alveolates and Rhizaria were more abundant in water and represented 28 and 24% of the total quantity, respectively (Figure 6A). The Stramenopiles were basically composed of diatoms (Bacillariophyceae) and other minor groups (Figure 6B), more abundant in sediment. The most abundant genera were *Navicula, Amphora, Talaroneis, Pseudo-nitzschia, Sellaphora, Thalassiosira, Thalassionema*, and *Alaucoseira* (Figure 7A). Brown algae (Phaeophyceae) of the genera *Ectocarpus, Myrionema, Battersia*, and *Sargassum* were abundant in the mesoplankton (Figure 7B).

The Alveolata group was mainly composed of dinoflagellates (Dinophyceae), followed by apicomplexan organisms and ciliates (Figure 6C). Apicomplexans of the genera *Selenidium* and similar to *Margolisiella* were also found in the top 25 most abundant protist groups, especially in sediment and microplankton (Figures 7A,C). Dinoflagellates were abundant in the environment, although the genus *Oxyrrhis* was the largest taxon in the nanoplankton. Other abundant genera were *Pentapharsodium, Ansanella, Blastodinium, Parvodinium*, and *Proterythropsis* (Figure 7D). Dinoflagellates that cause toxic blooms, such as *Amphidinium*, were detected in the sediment and nanoplankton. Other harmful dinoflagellates, such as *Alexandrium, Gymnodinium*, and *Prorocentrum*, were also detected, although they were not included in the top 25 most abundant genera.

Rhizaria was mainly composed of benthonic Foraminifera (*Ammonia*), Silicofilosea, Thecofilosea, and other minor organisms, such as cercomonads, Haplosporidia, and Vampyrellida (Figure 6D). The genera *Massisteria* and *Cercomonas* (Cercozoan), *Scutellomonas* and *Protapsis* (Thaumatomonadida), and *Rhogostoma* (Thecofilosea) were detected in water and sediment, but they were predominant in the microplankton (Figure 7C).

### Diversity of Potential Pathogens Found in the Ecosystem

A high number of potential pathogens belonging to several taxonomic groups were found in the ecosystem. Although some pathogens were included in the metazoan and fungi groups, the wide majority belonged to the protist group.

Pathogenic metazoans included several species of cnidarians, platyhelminths and arthropods that were mainly associated with water (Table 4). Platyhelminthes within the genus *Parvatrema*, as well as *Gymnophalloides seoi* and *Bucephalus minimus*, were the most abundant. The cnidarians (*Kudoa, Myxobolus*, and *Parvicapsula*) and the arthropod *Demodex folliculorum* were also detected (Table 4). The pathogenic fungi *Malassezia* and Chrytridiomycota were found in water and sediment (Table 4).

**Table 4:** Diversity of potential pathogens included in the metazoan and fungi taxonomic groups detected in the environment.

	**Organism**	**Sediment**	**Water**	**Hosts**	**References**
Cnidaria	*Kudoa unicapsula*	0	25	Several fish species	(50)
	*Myxobolus exiguus*	1	17	Myxobolids	(51)
	*Parvicapsula anisocaudata*	2	7	*Paralichthys olivaceus*	(52)
Platyhelminthes	*Acanthobothrium* sp.1	0	506	*Raja asterias*	(53)
	*Diplectanum aequans*	0	191	*Dicentrarchus labrax*	(54)
	*Parvatrema* sp. CG-2014	0	802	*Tagelus plebeius*	(55)
	*Gymnophalloides seoi*	0	610	Human intestinal parasite Oyster, second intermediate	(56)
	*Bucephalus minimus*	0	577	*Cerastoderma edule*	(57)
	*Prosorhynchoides borealis*	0	171	*Abra alba*	(58)
	*Paragonimus kellicotti*	1	153	Humans (consumption of undercooked crayfish meat)	(59)
	*Dicrogaster contracta*	0	107	*Chelon labrosus*	(60)
Arthropoda	*Demodex folliculorum*	5	42	Human skin and eyes	(61)
Basidiomycota	*Malassezia* sp.	156	1,091	Human skin	(62)

*Their abundance in sediment and water is presented. References supporting their pathogenicity in several hosts are also included. All organisms were confirmed with the 100% similarity with the NCBI database*.

The protists included the highest number of potential pathogens in a wide variety of marine organisms. Recognized pathogens are members of the Amoebozoa, Excavata, and TSAR supergroups, as detailed in Table 5. Pathogenic Amoebozoa of farmed fish and marine invertebrates, such as the genera *Paramoeba* and *Hartmannella*, were detected in water. Pathogens belonging to the Excavata group included the genera *Neobodo*, which are responsible for diseases in a wide range ascidian.

**Table 5:** Diversity of potential protist pathogens detected in the environment.

	**Phyla**	**Order**	**Genus**	**S**	**W**	**Host**	**Reference**
Amoebozoa		-	*Paramoeba*	141	694	Sea urchins. farmed fish, crustaceans	(63–65)
			*Hartmannella*	16	445	Rainbow trout	(66)
Excavata		Kinetoplastida	*Neobodo*	398	457	Ascidians	(67)
Stramenopiles	Oomycetes	Lagenidiales	*Halocrusticida*	343	1,191	Crustaceans	(68)
			*Lagenidium*	15	48	Fungi, algae, nematodes, rotifers, insects, crustaceans, mammals	(69–72)
		Myzocytiopsidales	*Eurychasma*	35	212	Brown algae	(73)
			*Myzocytiopsis*	27	117	Nematodes	(74–76)
		Olpidiopsidales	*Olpidiopsis*	5	290	Red, green and brown algae	(77)
		Peronosporales	*Phytophthora*	1	20	Terrestrial plants	(78)
		Pythiales	*Pythium*	19	901	Terrestrial plants, nematodes, insects, fish, mammals	(72, 79, 80)
		Saprolegniales	*Aphanomyces*	0	10	Crustacenas, fish	(81, 82)
	Labyrinthulomycetes	Labyrinthulaceae	*Labyrinthula*	8	99	Abalone, clams, flatworms, seastar, and eelgrass populations	(83–90)
			*Stellarchytrium*	16	44		
		Thraustochytriaceae	*Aplanochytrium*	453	6,646		
			*Oblongichytrium*	363	717		
			*Thraustochytrium*	1,001	2,826		
Alveolata	Apicomplexa	Archigregarinorida	*Selenidium*	2,523	4,484	Polichaeta, sipunculida and some hemichordate	(91)
	NA	Philasterida	*Pseudocohnilembus*	0	657	Olive flounder, rainbow trout	(92–95)
			*Cohnilembus*	13	2	Farmed fish	(96)
			*Miamiensis* *Philasterides*	12	68	Turbot, sharks	(40, 97–99)
			*Parauronema*	64	53	Farmed fish	(96)
	NA	Philasterida	*Philaster*	10	32	Corals (*Acropora muricata*)	(100)
			*Porpostoma*	22	11	Farmed fish	(96, 101)
			*Anophryoides*	7	2	Crustaceans (*Homarus americanus*)	(102, 103)
			*Metanophrys*	0	7	Farmed fish	(96)
		Syndiniales	*Amoebophrya*	28	882	Dinophyceae (including HA)	(104–106)
			*Duboscquella*	3	44	Ciliata (*Favella ehrenbergii, F. panamensis*)	(107, 108)
			*Hematodinium*	8	15	Marine crustaceans	(109, 110)
		Perkinsidae	*Parvilucifera*	11	163	Dinoflagellates	(111)
			*Perkinsus*	19	120	Bivalves	(34, 112)
		Suessiales	*Symbiodinium*	558	164	Anemones, sponges, mollusks, ciliates, foraminiferans	(113)
			*Pelagodinium*	18	223	Foraminifera	(114)
		–	*Cryptocaryon*	31	3	Marine fishes	(115, 116)
		Chlamydodontida	*Chilodonella*	0	15	Farmed freshwater fishes	(117–119)
Rhizaria	NA	Haplosporidia	*Haplosporidium*	0	12	Molluscs and crustaceans	(120–122)
		Imbricatea	*Pseudopirsonia*	98	1,031	Various diatom species	(123)
		Thecofilosea	*Rhogostoma*	13	1,439	Fish	(124)

The highest number of protists considered potential pathogens were included inside the different TSAR supergroups: Stramenopiles, Alveolata, and Rhizaria.

Pathogenic Stramenopiles were detected in sediment and water, and they include several members of the phyla Labyrinthulomycetes (*Aplanochytrium* and *Thraustochytrium)* and Oomycetes (*Halocrusticida*), which infect marine invertebrates. *Aplanochytrium, Thraustochytrium*, and *Halocrusticida* were in the top 25 most abundant protists in the marine environment (Figure 7). Low abundant genera of Oomycetes described as marine and terrestrial pathogens, such as Lagenidiales, Pythiales, Myzocytiopsidales, Peronosporales, Olpidiopsidales, and Saprolegniales, were also detected. The designated pathogen declared by the World Organization for Animal Health (O.I.E.), *Aphanomyces* (Saprolegniales), which is responsible for fish and crustacean diseases, was detected in the environment.

Pathogens included in the alveolates were mainly grouped in the orders Apicomplexa, Philasterida Syndinial, Perkinsidae, and Suessiales. Apicomplexans are parasites of marine organisms such as molluscs, polychaetes, fishes, and vertebrates, including domestic animals and even humans. Among all the apicomplexans, the genus *Selenidium* (Archigregarinorida) was the most abundant in sediment and microplankton (Figures 7A,C, Table 5) but other non-classified apicomplexans were also detected. The Philasterida order detected in the water was mainly associated with parasites of farmed fish. They included the genera *Pseudocohnilembus, Parauronema, Philasterides*/*Miamiensis, Cohnilembus, Anophyoides, Porpostoma, Phylaster, and Metanophrys* (Table 5). The genus *Amoebophrya* (Syndiniales), described as a pathogen in dinoflagellates, was dominant in water. *Hematodinium* and *Duboscquella* were also found, although in low abundance. *Perkinsus* (Perkinsidae), a O.I.E listed pathogen of mollusc was mainly detected in the water, but also in sediment. *Cryptocarion*, a well-known pathogen in marine fishes, was also present in sediment (Table 5).

Pathogens belonging to the rhizarians included the genus *Rhogostoma*, primarily associated with microplankton (Figure 7C). Moreover, the genus *Pseudopirsonia*, which affect diatoms and algae, was abundant. In lower abundance the genus *Haplosporidium* was also detected.

## Discussion

The “One Health” approach recognizes that human and animal health are interconnected within the ecosystem, especially in coastal marine environments, where human activities can dramatically alter microbial biodiversity (125). Marine infections have been reported in relation to cultured species or after massive mortalities of different species. As an example, coral diseases have received increasing attention because of the recent decline in coral reefs (126). However, although mass mortalities and disease outbreaks have been reported, it is difficult to understand the extent of these events. Moreover, we do not always know the causes or even if these diseases can be affected by anthropogenic effects. In recent years, concerns have risen regarding how human-mediated climate change can alter the composition of ecological communities and therefore modify pathogen abundance and patterns of disease transmission (127). Although many studies have been conducted in different parts of the world on marine biodiversity, the absence of an efficient and coordinated monitoring system could be a significant obstacle to our understanding of the pathogen distribution in the marine environment and the possible effects of anthropogenic activities, such as pollution and fishing. Global trade and travel can introduce new species, particularly in estuarine habitats, and successfully introduced pathogens could have broad host specificity and be pathogenic to new species.

One interesting result of our study was the identification of invasive species. Early detection of invasive species is essential for their management and to avoid the displacement of autochthonous species producing variations in the ecosystem (19). The European Union has ranked 267 marine species based on their likelihood of arrival, establishment, spread, and impact in EU waters (128). We detected four invasive species on the Galician coast, with the bivalve *Xenostrobus securis* being one of the most harmful invasive molluscs. The presence of *X. securis* on the Galician coast has been described (129), and its invasiveness and potential impact on autochthonous communities have already been evaluated (130, 131). In contrast, the microscopic arthropod *Pseudodiaptomus marinus* has not been previously reported, and its high abundance could suggest the presence of a stable population of this species in northwestern Spain between the previously recorded distribution areas of northern Europe and Mediterranean Sea (132) using a comparable metabarcoding approach (133). The appearance of previously non-reported organisms, could indicate that maritime transport (fouling and ballast water) plays a fundamental role in expanding the geographic distribution of exotic species.

Although our aim was mainly to obtain an overview of invasive and putative pathogenic species in the Ría de Vigo, and the study of V9 amplicons provides good resolution of protist diversity, our study also offers information on the presence and variety of fish species (134, 135). Metagenomics is being used as a less costly and faster monitoring tool of stocks based on the apparent good correlation between eDNA and fish biomass (136). Our results allowed the detection of 41 fish species grouped into 22 different orders, representing ~60% of the total orders previously described on the Galician coast (137). These results highlight the potential of eDNA metabarcoding to conduct a correct estimation of fish biodiversity, management of fisheries, including the detection of spawning seasons, regulation of protected spawning areas, etc., although this methodology requires a proper sampling and further validation (138). In this context, we detected a high prevalence of clupeids (*Clupea harengus*) and moronids (*Dicentrarchus punctatus*) during autumn and winter, corresponding with their spawning season (139).

The impact of harmful algal blooms (HABs) has increased in recent decades, and several management strategies have been adopted by countries to mitigate, prevent, and control HABs in marine waters (140). Their uncontrolled proliferation produces toxic and harmful effects on fish, shellfish, marine mammals, birds, and humans, affecting the aquacultural sector and human public health (141, 142). In this study, organisms included in the Taxonomic Reference List of Harmful Microalgae (IOC-UNESCO) (143) were very abundant, and some of them are responsible for producing toxins, such as the diatom *Pseudonitzschia* [that produces domoic acid neurotoxin (144, 145) and generates Amnesic Shelfish Poisining (ASP) outbreaks in Galician waters (146)] and the dinoflagellate *Amphidinium*, that contains the species *Amphidinium carterae* recognized to produce haemolytic substances and ichthyotoxins with harmful effects in invertebrates (147, 148).

Our work was conducted in an important area bivalve's production, therefore, the detection of potential pathogens of these animals could have a great impact on bivalve culture. This is the case of *Perkinsus olseni*, detected in sediment and planktonic fractions, which has been cataloged by the O.I.E. as a notifiable parasite that causes perkinsosis disease in bivalves, mainly in clams. *Haplosporidium* sp. was also found in our samplings, although at low abundance. This genus infects a wide range of marine invertebrates like oysters or mussels (122).

Our study also revealed pathogens that produce important diseases in fish, with a strong economic impact on aquaculture. The order Philasterida includes several pathogens that infect cultured flatfish, causing scuticociliatosis, one of the most critical parasitological diseases in marine aquaculture worldwide. This disease has led to severe economic losses, particularly in olive flounder and turbot aquaculture (40, 92, 98). Philasterida genera were detected in sediment and water in our study, and the genus *Pseudocohnilembus* was the most abundant in the water column. This species has been responsible for scuticociliatosis in the olive flounder *Paralichthys olivaceus* in Korea (93) and the rainbow trout *Oncorhynchus mykiss* in the United States (95). However, in Galicia, scuticociliatosis has been mainly attributed to *Philasterides dicentrarchi* or *Miamiensis avidus* (40, 98), which were also detected in sediment and water fractions but in lower abundance than *Pseudocohnilembus*.

Another important group of fish pathogens that were identified in our work were those causing Nodular Gill Disease (NGD) in salmonids in Europe (66, 124, 149). The protist *Rhogostoma*, highly detected in water, has been associated with NGD, in particular, the species *Rhogostoma minus*, which induces hyperplasia of gill lamellae and, as a consequence, is one of the most critical fish disorders (149). Other abundant pathogenic genera, such as *Paramoeba* and *Hartmanella*, including harmful amoeba species, were detected in the sediment and water column. These species have been associated with NGD in freshwater fish and with Amoebic Gill Disease (AGD) in other species, such as Atlantic salmon (*Salmo salar*), rainbow trout (*Oncorhynchus mykiss*), brown trout (*Salmo trutta*) or turbot (*Scophthalmus maximus*) (150). Moreover, although *Nolandella*, the most abundant amoeba genus in our study, does not seem to be pathogenic, it has been reported to colonize the gills of farmed Atlantic salmon with AGD (151).

Putative pathogens can play essential functions in the ecosystem or affect other species without commercial interest but can also be valuable for the health of the ecosystem. Apicomplexans such as gregarines are still poorly studied and restricted to invertebrate hosts (152). Nevertheless, Archigregarine *Selenidium* was one of the most abundant protists in sediment and microplankton. This parasite infects the intestine of marine invertebrates, such as Polichaeta, Sipunculida, and some Hemichordata, explaining their high abundance in water but also in sediment (91, 153). Other gregarines from the families Lecudinidae and Eugregaronida were detected in the environment. These organisms infect invertebrates, including polychaetes, crustaceans or bivalves (152, 154); however, available information on gregarines in Spain is limited (155), therefore an accurate classification of organisms from this group at genus level would be necessary with the combination of other molecular techniques. These parasites have an important impact on invertebrates, such as crustaceans disrupting the ecosystem, and have been described as affecting shrimp aquaculture (156).

The oomycete *Aphanomyces astaci*, an organism native to North America, is also cataloged as a notifiable pathogen by the O.I.E. This organism infects aquatic decapods, and has been responsible for severe mortalities of native crayfish species in Europe in which their populations have declined (82). Although their abundance was very low in the environment, future surveillance of this pathogen should be performed in the study area to avoid possible propagation of this pathogen.

Oomycetes and Labyrithulomycetes infect many marine invertebrates (68, 157) and microalgae, even those involved in harmful algae blooms (73, 158). Our results showed a high abundance of Lagenidiales, which are parasites in several crustaceans and microalgal genera (69, 73, 157). Labyrinthulomycetes are opportunistic pathogens in bivalves, clams, and flatworms, and they cause severe diseases in eelgrass populations (83).

Planktonic diatoms, one of the main dominant groups in our metagenomic study, are susceptible to infections caused by parasitoids such as the rhizarian *Pseudopirsonia*, which it is very abundant in the environment (123, 159, 160). They are also affected by fungal organisms belonging to the Chitridiomycota group (161), detected mainly in sediment, and previously described as dominant in the nearshore and sediment samples (162).

Our metabarcoding study also allowed us to detect pathogens in mammals, confirming that human and animal health are interconnected within the marine ecosystem. The phylum Apicomplexa, as in previous studies such as the Tara Ocean expedition (163), was highly represented in the Ría de Vigo. Apicomplexan parasites are known to infect a wide range of animals, including humans and domestic animals with medical and veterinary importance worldwide, and include *Toxoplasma* (164), *Cryptosporidium* (165) and *Babesia* (166). In our study, pathogens similar to *Cryptosporidium* were amply detected in sediment and planktonic fractions, however, further analysis should be done to clearly identify these organisms because these parasites, with a waterborne transmission, have been associated with economic losses in animals such as ruminants (167). Transmissible stages are ubiquitous in aquatic habitats such as irrigation water, recreational areas, and wastewater and drinking water treatment plants. This spread could explain the high abundance obtained in planktonic fractions, according to its previous detection in Galicia (167, 168).

In conclusion, the metagenomic assay of the V9 region of the 18S SSU rRNA gene has provided valuable information on the eukaryotic composition in Ría de Vigo, and the identification of several pathogenic organisms included protists, fungi, and metazoans. The presence of specific genera and species should be validated in the future by using other techniques and taking into account the gene copy number normalization (169) and even the bias produced by the amplicon PCR but the metabarcoding method allowed us to establish a baseline of putative invasive and pathogenic organisms in this marine ecosystem.

Ría de Vigo is known for its high fish and shellfish productivity and diversity dynamics, especially during upwelling periods. The release of pollutants to the marine ecosystem, the movement of species from other areas or the introduction of foreign species by ballast water, could be factors that could change the composition of the ecosystem in Ría de Vigo in the future and benefit the uncontrolled proliferation of the known eukaryotic pathogens, causing severe mortalities to species with a high importance in the aquaculture sector. For these reasons, the use of metabarcoding assays for the monitoring of potential pathogens, invasive species, and the causative agents of harmful phytoplanktonic blooms could be important to evaluate the health of a marine ecosystem that directly affects the aquacultural sector and even human and veterinary health.

## Data Availability Statement

The data presented in the study are deposited in the Sequence Read Archive (SRA) (http://www.ncbi.nlm.nih.gov/sra), accession number PRJNA761019.

## Author Contributions

BN and AF conceived and designed the project. RR-C, AR, and RA conducted the analyses. AP, AF, RR-C, and EB performed the data analysis and the bioinformatic studies. RR-C wrote the manuscript. All authors contributed to the article and approved the submitted version.

## Funding

This work was conducted with the support of the projects AGL2015-65705-R (Ministerio de Economía y Competitividad, Spain), IN607B 2019/01 (Consellería de Economía, Emprego e Industria–GAIN, Xunta de Galicia), 0474_BLUEBIOLAB Fondo Europeo de Desarrollo Regional FEDER en el marco del programa Interreg V A España – Portugal (POCTEP) 2014-2020, and VIVALDI (678589) (EU H2O20). RR-C wishes to thank the Axencia Galega de Innovación (GAIN, Xunta de Galicia) for her predoctoral contract IN606A-2018/020.

## Conflict of Interest

The authors declare that the research was conducted in the absence of any commercial or financial relationships that could be construed as a potential conflict of interest.

## Publisher's Note

All claims expressed in this article are solely those of the authors and do not necessarily represent those of their affiliated organizations, or those of the publisher, the editors and the reviewers. Any product that may be evaluated in this article, or claim that may be made by its manufacturer, is not guaranteed or endorsed by the publisher.
