# Supplementary material for: High-Throughput Sequencing of Environmental DNA as a Tool for Monitoring Eukaryotic Communities and Potential Pathogens in a Coastal Upwelling Ecosystem
Source: Front Vet Sci. 2021 Nov 3;8:765606. doi: 10.3389/fvets.2021.765606 (PMC8595318; doi:10.3389/fvets.2021.765606)
Supplement: Supplementary file 1 [file Table_1.DOCX]

**Supplementary Table 1:** Number of reads before and after trimming for individual samples. The total abundance of reads is also indicated.

|  | **Sample** | **Total reads** | **Reads after**  **trimming** | **% trimmed** | **av lenght**  **after trimming** | **Total**  **abundance** |
| --- | --- | --- | --- | --- | --- | --- |
| Summer 2016 | Sediment | 161.074 | 154.699 | 96.04% | 138 | 67336 |
|  | Meso- | 204.766 | 192.841 | 94.18% | 137 | 88836 |
|  | Nano- | 209.264 | 192.735 | 92.10% | 136 | 74029 |
| Autumn 2016 | Sediment | 196.382 | 147.202 | 74.96% | 135 | 50627 |
|  | Micro- | 207.128 | 190.949 | 92.19% | 133 | 82182 |
|  | Nano- | 187.606 | 157.932 | 84.18% | 135 | 68834 |
| Winter 2017 | Sediment | 176.332 | 158.73 | 90.02% | 135 | 60529 |
|  | Meso- | 187.586 | 178.256 | 95.03% | 136 | 80551 |
|  | Micro- | 199.924 | 166.504 | 83.28% | 136 | 75347 |
| Summer 2017 | Sediment | 199.928 | 190.154 | 95.11% | 136 | 73241 |
|  | Meso- | 171.286 | 160.837 | 93.90% | 138 | 62861 |
|  | Micro- | 185.474 | 176.238 | 95.02% | 136 | 79816 |
|  | Nano- | 190.22 | 131.551 | 69.16% | 137 | 55955 |
| Autumn 2017 | Sediment | 220.044 | 188.067 | 85.47% | 136 | 80395 |
|  | Micro- | 219.086 | 103.173 | 47.09% | 136 | 46108 |
|  | Nano- | 212.182 | 195.913 | 92.33% | 132 | 88888 |
| Winter 2018 | Sediment | 136.896 | 132.413 | 96.73% | 138 | 56656 |
|  | Meso- | 201.416 | 188.426 | 93.55% | 140 | 83233 |
|  | Micro- | 225.334 | 211.181 | 93.72% | 135 | 90508 |
|  | Nano- | 214.094 | 160.721 | 75.07% | 135 | 71929 |
| Summer 2018 | Sediment | 181.864 | 174.603 | 96.01% | 136 | 70796 |
|  | Meso- | 191.408 | 188.402 | 98.43% | 140 | 90311 |
|  | Micro- | 203.386 | 132.79 | 65.29% | 136 | 58854 |
|  | Nano- | 200.978 | 160.496 | 79.86% | 135 | 71183 |
